# Glucocorticoid agonists enhance retinal stem cell self-renewal and proliferation

**DOI:** 10.1186/s13287-021-02136-9

**Published:** 2021-01-25

**Authors:** Kenneth N. Grisé, Nelson X. Bautista, Krystal Jacques, Brenda L. K. Coles, Derek van der Kooy

**Affiliations:** 1 Department of Molecular Genetics, University of Toronto, Toronto, ON M5S 1A8 Canada; 2 Donnelly Centre for Cellular and Biomolecular Research, University of Toronto, Toronto, ON M5S 3E1 Canada; 3 Institute of Medical Science, University of Toronto, Toronto, ON M5S 1A8 Canada

**Keywords:** Stem cell, progenitor, Retina, Glucocorticoid, proliferation, Self-renewal, Photoreceptor, Neurogenesis, Cell differentiation, Drug discovery, Dexamethasone

## Abstract

**Background:**

Adult mammalian retinal stem cells (RSCs) readily proliferate, self-renew, and generate progeny that differentiate into all retinal cell types in vitro. RSC-derived progeny can be induced to differentiate into photoreceptors, making them a potential source for retinal cell transplant therapies. Despite their proliferative propensity in vitro, RSCs in the adult mammalian eye do not proliferate and do not have a regenerative response to injury. Thus, identifying and modulating the mechanisms that regulate RSC proliferation may enhance the capacity to produce RSC-derived progeny in vitro and enable RSC activation in vivo*.*

**Methods:**

Here, we used medium-throughput screening to identify small molecules that can expand the number of RSCs and their progeny in culture. In vitro differentiation assays were used to assess the effects of synthetic glucocorticoid agonist dexamethasone on RSC-derived progenitor cell fate. Intravitreal injections of dexamethasone into adult mouse eyes were used to investigate the effects on endogenous RSCs.

**Results:**

We discovered that high-affinity synthetic glucocorticoid agonists increase RSC self-renewal and increase retinal progenitor proliferation up to 6-fold without influencing their differentiation in vitro. Intravitreal injection of synthetic glucocorticoid agonist dexamethasone induced in vivo proliferation in the ciliary epithelium—the niche in which adult RSCs reside.

**Conclusions:**

Together, our results identify glucocorticoids as novel regulators of retinal stem and progenitor cell proliferation in culture and provide evidence that GCs may activate endogenous RSCs.

**Supplementary Information:**

The online version contains supplementary material available at 10.1186/s13287-021-02136-9.

## Background

Retinal degenerative diseases cause permanent vision loss in mammals because the retinal neurons that are lost, such as photoreceptors and retinal ganglion cells (RGCs), are not replaced and their axons do not regenerate after damage [1, 2]. Stem cells and embryonic retinal progenitors have been used as exogenous cell sources for retinal transplantation therapies with varying degrees of efficacy depending on the cell source, the degree of pre-transplant cell type maturity, and the number of cells transplanted [3]. However, to what degree the functional improvement from transplanted cells is due to the secretion of trophic factors or material transfer of phototransduction machinery is still being actively investigated [3]. Advances in stem cell biology have heralded activation of resident adult stem cells as a promising strategy for tissue regeneration [4–6]. In the mammalian eye, Müller glia (MG) [7, 8], the retinal pigmented epithelium (RPE) [9, 10], and the ciliary epithelium (CE) [11, 12] have been identified as tissues containing a subset of cells with stem/progenitor-like properties and retinal neurogenic potential. Thus, whether these cells can be sources for endogenous retinal regeneration is actively being investigated.

Retinal stem cells (RSCs) are a rare and quiescent subpopulation of cells in the pigmented layer of the CE of the mammalian eye that are capable of clonal expansion, self-renewal, and differentiation into all the cell types of the retina when isolated in vitro [11–15]. RSCs in the mammalian CE have been compared to the proliferative ciliary marginal zone (CMZ) of non-mammalian vertebrates which harbor stem cells that have neurogenic and regenerative potential in the adult eye [16]. Recent in vivo lineage tracing studies have shown that CE progenitor cells migrate into the peripheral retina and generate all seven major retinal cell types during eye development [17, 18], similar to CMZ progenitors [5, 16]. However, unlike the CMZ, that process arrests postnatally, and no further generation of retinal neurons by the CE is observed. Despite the expression of stem cell and retinal progenitor genes in CE-RSCs and their progeny, some studies have suggested that they are not true stem cells based on the observation of a limited in vitro proliferative/self-renewal ability, maintenance of features of epithelial cells in RSC progeny, and described only ectopic expression of mature retinal cell markers after differentiation [19, 20], suggesting that CE cells might have general proliferative competency and plasticity as opposed to containing rare stem cells. However, the ability to prospectively identify and sort RSCs indicates a pre-existing rare cell type within the CE with proliferative competency [15], while in vitro growth and self-renewal of RSCs and their progeny can be profoundly enhanced based on cell culture conditions [13, 21]. Further, RSC-derived photoreceptors have been shown to be functional pre-transplant in vitro [22, 23], as well as post-transplant in vivo [24]. Thus, CE RSCs continue to be investigated as an exogenous source for cell replacement therapy and a potential source of endogenous retinal regeneration [5, 25] However, attempts to activate adult mammalian RSCs in vivo have not been effective, an outcome that is attributed to the presence of quiescence factors in the RSC niche [26, 27].

In this study, we sought to further elucidate the mechanisms that regulate RSC proliferation by screening small molecules with known molecular targets and identifying the compounds that stimulate retinal stem and progenitor cell (RSPC) proliferation. We discovered that synthetic glucocorticoid (GC) agonists enhance the proliferation of retinal progenitors and increase the symmetric self-renewal of RSCs in culture. Furthermore, intravitreal injection of the synthetic GC agonist dexamethasone (Dex) into the adult mouse eye induced proliferation in the CE, the niche in which endogenous RSCs reside, indicating glucocorticoid signaling may stimulate RSC proliferation in vivo.

## Methods

### Mice

All mouse protocols were approved by the Animal Care Committee at the University of Toronto, which operates in accordance with the Canadian Council on Animal Care. Adult mice used in this study were a minimum of 8–10 weeks old including the following: CD1 mice (022, Charles River), C57/BL6J mice (000664, Jackson Laboratories), Actin-GFP mice [FVB.Cg-Tg (CAG-EGFP)B5Nagy/J; 003516, Jackson Laboratories], and mouse insulin promoter (MIP)-GFP mice [CD1/Tg (Ins1-EGFP/GH1); 006864, Jackson Laboratories]. Mice were kept on a 12-h light-dark/light cycle. Food was available ad libitum. Water was supplied ad libitum except during EdU delivery.

### Isolation of retinal stem cells from the ciliary epithelium of the adult eye and primary clonal sphere assay

A dissecting microscope, cold light source, and sterile surgical instruments were set up inside of a sterile biological safety cabinet (BSC). Mouse eyes were enucleated immediately prior to beginning the dissection protocol. Mouse eyes were placed in a petri dish containing cold, sterile regular aCSF. Under the dissecting microscope, the hair, connective tissue, and the dorsal and ventral oblique muscles were cleared from the scleral/corneal border with two sets of forceps. Next, curved or angled micro-dissecting scissors were used to cleave off any remaining extraocular muscle tissue and the optic nerve and cut the eyeball into symmetrical halves, beginning and finishing the cut from the hole left by the optic nerve. Using two sets of forceps to grasp the cornea, the two eye halves were peeled apart. The lens, retina, and vitreous were separated from the eye shells, and the eye shells were transferred into a new petri dish (also containing cold, sterile regular aCSF). To isolate the ciliary epithelium (CE), eye shells were oriented with the cornea on the right and retinal pigmented epithelium (RPE) on the left. A pair of straight forceps were used to pin down the eye shell on the RPE side while a scalpel blade was inserted between the CE and the iris, using pressure to slice the iris/cornea side off from the rest of the shell. Next, the scalpel was run along the border between the CE and the RPE to obtain the CE isolated as a thin strip of tissue. The CE strips were then transferred to a 35-mm dish containing 2 mL of dispase solution (Sigma; T1005) and incubated for 10 min at 37 °C. Next, the strips were transferred from dispase into a 35-mm dish containing 2 mL of sterile filtered kynurenic acid (02.mg/mL; Sigma), trypsin (1.33 mg/mL; Sigma), and hyaluronidase (0.67 mg/mL; Sigma) in high-magnesium/low-calcium artificial cerebral spinal fluid (hi/lo aCSF) and incubated at 37 °C for 10 min. After incubation, the dish was returned to the dissecting scope, and the CE strips were pinned down with straight, non-serrated forceps, while non-serrated curved forceps were used to scrape the CE off from the underlying sclera. The bare scleral strips were then discarded, such that only the CE cells remained in the enzyme solution. Using a fire-polished, cotton-plugged glass pipette, the cells and enzyme solution were transferred to a 15-mL tube and triturated approximately 45 times to break apart the tissue. The 15-mL tube with cell suspension was centrifuged for 5 min at 1500 rpm. The supernatant was gently aspirated from the resulting pellet using a fire-polished, cotton-plugged glass pipette, and 2 mL of sterile-filtered ovomucoid trypsin inhibitor (1 mg/mL; Sigma) in SFM was added to the pellet. Using a small borehole, fire-polished, cotton-plugged glass pipette, the sample was triturated approximately 45 times to generate a single-cell suspension. The 15-mL tube with cell suspension was centrifuged for 5 min at 1500 rpm. The supernatant was gently aspirated from the resulting pellet, and 1–2 mL of SFM with fibroblast growth factor 2 (FGF2, 10 ng/mL; Sigma) and heparin (2 μg/mL; Sigma) was added. The cells and media were mixed to ensure a uniform cell suspension, and a 10-μL sample was taken for cell density determination. The cells were then seeded and cultured at 10 cells/μL in culture-treated plates or flasks and incubated in a humidified incubator at 37 °C in 5% CO_2_ and ambient room O_2_. After 1 week, roughly 1 in 500 cells are expected to proliferate to form free-floating, clonal spheres greater than 80 μm in diameter.

### Mouse pancreatic multipotent progenitor isolation and sphere assay

A modified version of our previously described mouse pancreatic islet isolation protocol was performed [28]. Briefly, mice were anesthetized using sodium pentobarbital prior to terminal dissections; 1 mg/mL of collagenase type V (Sigma) dissolved in 1× HBSS (Gibco) was perfused through the bile duct. The perfused pancreas was incubated in a 37 °C water bath to digest the pancreas. The islets were immediately hand-picked out of the total pancreatic tissue. A pure population of islets was incubated with trypsin ETDA (Sigma) at 37 °C and triturated with a small-borehole siliconized pipette into a single cell suspension. Viable cells were counted using Trypan Blue (Sigma) exclusion, and the pancreatic multipotent progenitor (PMP) sphere formation assay was performed as previously described (Seaberg et al. [28]), with the addition of conditions containing dexamethasone at 0.1 μM, 1 μM, or 10 μM. PMP spheres were obtained from adult mice with an age ranging from 4 weeks to > 18 months from pooled sexes. PMP spheres derived from mouse insulin promoter (MIP)-GFP mice [CD1/Tg (Ins1-EGFP/GH1); 006864, Jackson Laboratories] were used for live sphere quantification of insulin-GFP intensity. Similar sized spheres from SFM, DMSO, and 1 μM Dex conditions were pulsed with Hoechst for 30 min, and 8–10 confocal *z*-stack images were taken with GFP, and DAPI channel power remaining constant for all images. Projection images were created and analyzed on ImageJ (https://imagej.nih.gov/ij/) by finding the average pixel intensity value for each sphere and comparing the means of spheres in each condition. More specifically, TIF images of only the GFP channel of each sphere were imported into ImageJ and converted to grayscale (16 bit). Individual spheres were isolated by tracing around the sphere border, and the mean gray value was extracted for quantitative analysis.

### Sphere passaging

RSC spheres were passaged using hyaluronidase (0.67 mg/mL), collagenase I (0.5 mg/mL), and collagenase II (0.5 mg/mL) dissolved in Accustase solution (Sigma; SCR005). Spheres were collected en masse from culture plates or flasks, transferred into one or more 50-mL tubes, and centrifuged for 5 min at 1500 rpm. The supernatant was gently aspirated from the pellet, and 2–5 mL of enzyme solution was added to the pellet and mixed thoroughly. The 2–5-mL enzyme and sphere suspension was transferred to a 15-mL tube and laid horizontally on an automated rocker at 37 °C for 45 min. After incubation, the enzyme solution with spheres was triturated approximately 45 times to mechanically dissociate the spheres. The cell suspension was centrifuged for 5 min at 1500 rpm. The supernatant was gently aspirated, and 1–2 mL of trypsin inhibitor solution was added to the pellet and triturated approximately 45 times. The cell suspension was centrifuged for 5 min at 1500 rpm. The supernatant was gently aspirated from the resulting pellet, and 1–2 mL of SFM with FGF2 and heparin (plating media) was added. The cells and media were mixed to ensure a uniform cell suspension, and a 10-μL sample was taken and cell density was determined from that sample. The main cell pellet was then diluted to 10 c/μL.

### Medium-throughput screening pipeline

The 400-compound Ontario Institute for Cancer Research (OICR) Tool Compound Library (TCL; see Supporting Information) 1-mM stock plate, screening plate preparation, and cell seeding apparatus were provided by the Toronto Hospital for Sick Children SPARC Biocentre (Toronto, ON). An Echo acoustic dispenser (Labcyte) was used to seed 100 nL of DMSO into all vehicle control wells of five 96-well assay plates. Next, 100 nL of the 1-mM OICR TCL plate drugs were transferred according to the predetermined plate map layouts into the assay plates (see Supporting Information for plate map). Actin-GFP mouse primary RSC spheres were grown and then passaged into a single cell suspension of secondary RSPCs at a density of 10 cells/μL, according to the sphere growth and passaging methods detailed above. Next, 100 μL of cells was seeded into each well of the prepared assay plates using the Bravo liquid handler (Agilent) to result in 1000 cells/well, with drug wells at 1 μM and all wells at 0.1% DMSO. After seeding, another 100 μL of cells with 0.1% DMSO was added to the 2× pseudo-positive control wells to achieve a final density of 2000 cells/well. The assay plates were then incubated at 37 °C for 7 days. On day 7 of the MTS assay, Hoechst 33342 (10 μg/mL; Thermo Fisher) was added directly to each well of the 96-well plates, and cells were imaged (according to medium-throughput and medium-content imaging detailed below) for a minimum of 10 min afterward.

### Medium-throughput and medium-content imaging

At primary screening, 96-well plate imaging (2D culture) was performed using a Celigo imaging cytometer, equipped with × 4 F-theta lens and 2024 × 2024 CCD camera (Nexcelom Bioscience). The Celigo software suite was used to extract cell counts and area quantifications for cells that were grown for 1 week and then live stained for the nuclei (Hoechst 33258; 10 μg/mL). An actin-GFP transgenic mouse strain was used, and cell number quantifications were made based on individual nuclei count in the DAPI channel or the total area of GFP-expressing cells.

Sphere assays in 24-well plates (3D culture) were imaged using IN Cell Analyzer 6000 (GE Healthcare) equipped with Nikon Plan Apo 4×/NA 0.2 objective and 2048 × 2048 sCMOS camera. 3D datasets (5 *z*-planes, 15-μm spacing) were acquired using the FITC channel for a total of 12 fields per well. *z*-stack was collapsed using maximum intensity projection and analyzed using a custom image analysis routine for MATLAB 2015b (MathWorks).

Live-dead assays and EdU proliferation assays in 24-well plates (2D culture) were also imaged using IN Cell Analyzer 6000 using the same objective and camera as for sphere assay described above. Fixed cells were stained with Hoechst 33258 to label all the nuclei and compared to the EthD-1- or EdU-positive nuclei. Image analysis to extract the total number of cells and number of EthD-1- or EdU-positive cells was performed in MATLAB 2015b (MathWorks) using a custom image analysis routine.

### Proliferation and cell death assays

24-well plates were coated with laminin (300 μL/well; Sigma) and incubated at least 4 h at 37 °C, then washed with SFM or PBS prior to cell seeding. Secondary RSPCs in a single cell suspension were seeded manually at 2500 cells/well (5 cells/μL). For cell death assays, 2 μM ethidium homodimer (EthD-1; Abcam ab145323) was added to cells and incubated for 15 min at 37 °C. EthD-1 was then washed out with 3 successive PBS rinses, and then cells were fixed using 4% paraformaldehyde (PFA, Sigma). For cell proliferation assays, 10 μM 5-ethynyl-2′-deoxyuridine (EdU; Sigma) was added to the cells and incubated for 3 h at 37 °C. EdU was then washed out with 3 successive PBS rinses, and then cells were fixed using 4% PFA. Fluorescent EdU labeling was achieved using the EdU Click-iT detection kit (Thermo Fisher). Hoechst (10 μg/mL) was added, and 24-well plates were imaged using the IN Cell Analyzer 6000 (GE Healthcare) to determine the total nuclei number vs the number of nuclei labeled by EthD-1 or EdU (see “High-throughput and high-content imaging”).

### Differentiation assay

24-well plates were coated with laminin (300 μL/well; Sigma) and incubated at least 4 h at 37 °C, then washed with SFM or PBS prior to sphere plating. Primary RSC spheres were picked using a 200-μL pipet, from plates on an inverted microscope with an external cold light source. Spheres were plated into wells pre-filled with 500 μL one of 3 treatment conditions: SFM+1%FBS, SFM+1%FBS+0.1% DMSO, or SFM+1%FBS+0.1% DMSO+1μM Dex. Two spheres per well were plated to prevent loss of wells as spheres occasionally do not fully adhere prior to the first media change. Media changes were performed every 4 days by aspirating old media and then refilling wells with 500 μL of the same treatment conditions. After 6 weeks, wells were washed with PBS and fixed with 4% PFA, and ICC was performed for retinal cell type markers.

### Immunohistochemistry and immunocytochemistry

Mice were euthanized by cervical dislocation while under isoflurane anesthesia. The eyeballs were enucleated from adult mouse skulls, postfixed in 4% PFA for 4 h at 4 °C, then transferred to a cryoprotectant 30% sucrose solution for a minimum of 24 h. Next, the eyes were embedded in Tissue Tek, frozen at − 80 °C, and then sectioned at 10 μm using a cryostat. Fixed frozen eye slides or fixed cells in wells were permeabilized with 0.3% Triton X-100 (Sigma) in PBS for 20 min. Then, they were blocked in 10% normal goat serum (NGS) or 10% normal donkey serum (NDS) for 1 h. Primary antibodies were diluted in 1% serum from the species used for blocking (to the dilution indicated below) and incubated overnight at 4 °C. After washing, secondary antibodies were diluted in 1% serum of the same species at 1:400 (Alexa Fluor, Thermo Fisher) and incubated for 1 h. After washing, the nuclei were stained with Hoechst 33258 (10 μg/mL) for 20 min before the final wash. A mounting medium was added to the wells or slides, and the slides were then coverslipped. Primary antibodies used in this study include rabbit anti-cone arrestin (1:1000; AB15282, Millipore), mouse anti-rhodopsin (1:500; MAB5316, Millipore), goat anti-calbindin (1:500; SC-7691, Santa Cruz), rabbit anti-PKCα (1:1000; P4334, Sigma), mouse anti-syntaxin (1:100; AB3265, Abcam), goat anti-Brn3a (1:500; SC-31984, Santa Cruz), mouse anti-GFAP (1:500; G3893, Sigma), mouse anti-RPE65 (1:250; MAB5428, Millipore), rabbit anti-Pax6 (1:1000; AB2237, Millipore), rabbit anti-ERG (1:250; AB92513, Abcam), and rat anti-CD68 (1:500; MCA1957, BioRad).

### Intravitreal injections

Intravitreal injections were carried out using a 10-μL WPI Nanofil® Injector System with a micro-machined 34-gauge beveled needle (World Precision Instruments, Sarasota, FL), a dissecting microscope or surgical scope (Moller Hi-R 900C), a mouse stereotaxic apparatus, and a heat pad. Mice were brought to a surgical plane of anesthesia via 5% isoflurane and placed on the heat pad in the mouse stereotaxic apparatus (without head stabilization with the ear bars). Once anesthetized, isoflurane was reduced to 3% for maintenance. Mice were injected with 2 mg/kg meloxicam for analgesia. One drop of anticholinergic mydriatic (Mydriacyl®) was applied to each mouse eye to dilate the pupils. Mice were positioned on one side, so that the eye to be operated on was facing upward, directly under the surgical microscope. A small rubber washer was placed over the eye, so that the washer surrounds the eye like a monocle. A single drop of 3% methylcellulose (MC) solution (in saline) into the monocle, which allows clear visualization of the posterior segment of the eye by the surgeon. The mouse head was stabilized with the non-dominant hand, and the needle was controlled with the dominant hand. With the needle, a trans-scleral puncture was made (at a perpendicular angle to the globe) approximately 1 mm posterior of the limbus, in the nasal (anterior) aspect of the eye. The needle passed through the sclera, choroid, and retina to enter the retrolental vitreous. The needle was inserted as far as the central area of the retina, taking care to avoid striking the lens, retina, or the hyaloid canal. A 2-μL bolus of fluid was then injected at an approximate rate of 4 μL/min. The adult mouse vitreous space can accommodate up to 3 μL of total fluid because it replaces the fluid initially lost from the pre-injection vitreous outflow. Thus, the final vitreous volume in the eye is the same as the standard vitreous volume of the mouse eye (7 μL) [29]. Due to the expected final volume of 7 μL in the vitreous, Dex concentrations of 0.35 μM, 3.5 μM, and 35 μM were injected and in order to achieve a final concentration of 0.1 μM, 1 μM, and 10 μM in vivo. Once the injection was completed, the needle remained in the retrolental vitreous for an additional 10–15 s. This allows for pressure equilibration and works to prevent significant backflow following the withdrawal of the needle. Next, the needle was removed, the monocle was removed, and the mouse is rotated to position the other eye for surgery. Once the surgery on both eyes was completed, the mouse was left to recover alone in a recovery cage with a heat lamp and then reunited with its original cage-mates.

### Statistical analysis

Data are presented as mean ± standard error (SE) unless otherwise noted. Microsoft Excel was used to compute the strictly standardized mean difference (SSMD), signal-to-noise ratio, coefficient of variation, and hypergeometric test. All other statistical analyses were run using Sigmaplot 12 (Systat Software Inc.) or GraphPad Prism 6 (GraphPad Software Inc.). Student’s *t* test (two-tailed) was performed for statistical analysis between two groups. One-way ANOVA or a two-way ANOVA (for factor comparisons) with a Holm-Sidak or Fisher’s LSD multiple comparison post hoc test was used when three or more groups were compared. Sample size (*N*) values are provided in the figure legends. Statistical significance was set at *p* < 0.05.

## Results

### Medium-throughput screening identifies several unique compound classes that expand retinal stem and progenitor cells in culture

**Fig. 1:**
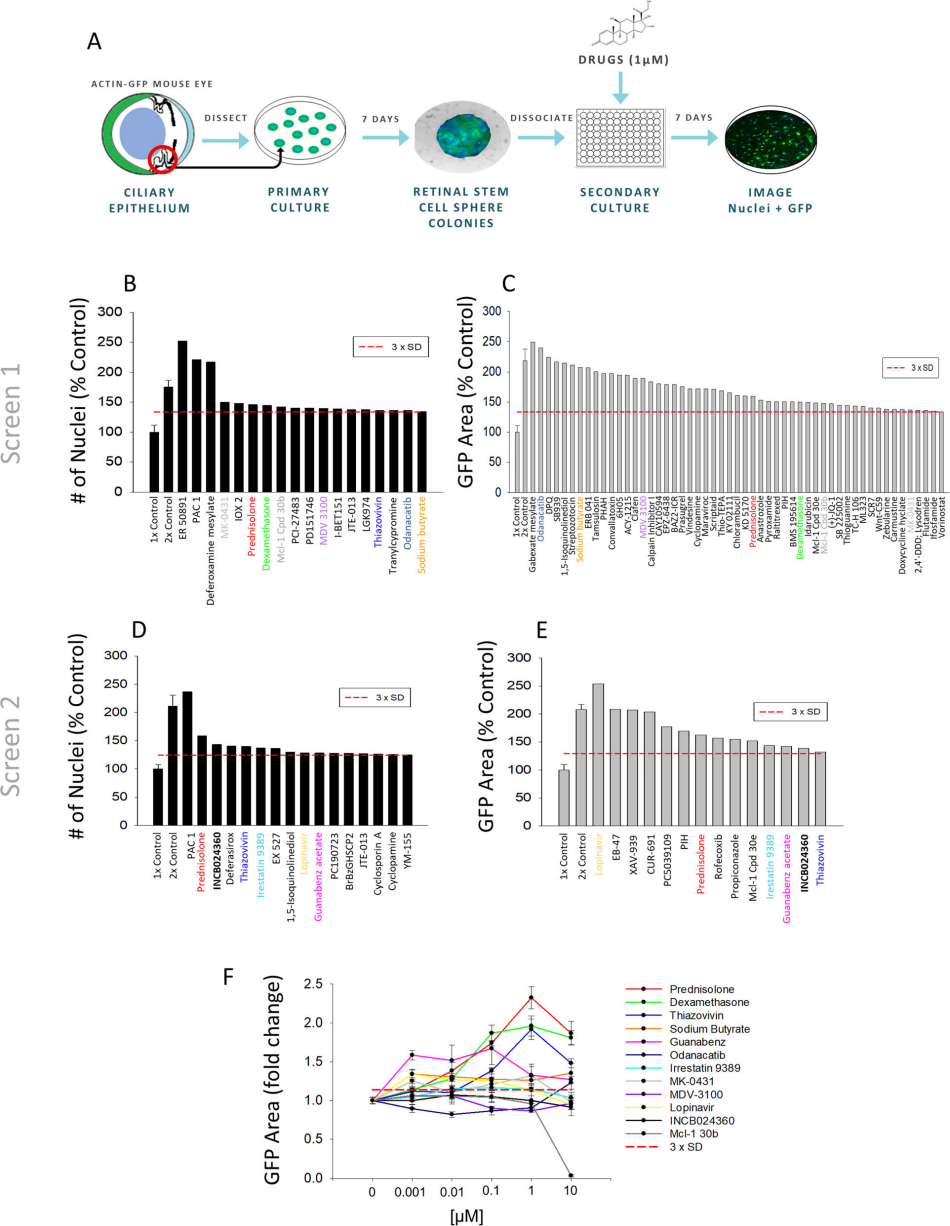
Medium-throughput screening identifies several unique compound classes that increase retinal stem and progenitor cell number. **a** Schematic overview of the MTS pipeline to detect RSPC expanding compounds. **b**–**e** Quantification as a percent of control (POC) of all compounds that met the hit criteria of having a relative number of nuclei or total area of actin-GFP expression over 3xSD above the 1× control mean. Hits that were identified in multiple criteria and/or screens are color-coded. **b**, **c** Seven compounds in screen 1 were found to be hits in both the number of nuclei and actin-GFP area and were selected as lead compounds. 1× control: *N* = 80 technical replicates. 2× control: *N* = 12 technical replicates. Compounds: *N* = 1 technical replicate. **d**, **e** Eight compounds in screen 2 were found to be hits in both the number of nuclei and actin-GFP area and were selected as lead compounds. 1× control: *N* = 80 technical replicates. 2× control: *N* = 15 technical replicates. Compounds: *N* = 1 technical replicate. **f** A dose-response assay of the hit compounds identified via MTS. *N* = 3 for all compounds at each concentration. Data are mean ± SEM

To identify compounds that can expand retinal stem and progenitor cell number, we developed a medium-throughput screening (MTS) pipeline that combined a method for the generation and seeding of retinal stem and progenitor cells with medium-throughput image analysis. Because RSC spheres contain varying proportions of pigmented cells that can obfuscate fluorescence, to help facilitate imaging, we used an albino actin-GFP mouse strain for primary RSC dissection and clonal sphere expansion. RSC primary spheres—which contain < 1% RSCs and over 99% retinal progenitor cells—were generated using a clonal sphere-forming assay [11, 26]. Cultures derived from RSC spheres are referred to as retinal stem/progenitor cell (RSPC) cultures. The RSC primary spheres were collected and dissociated into a single cell suspension (10 cells/μL, 1000 cells/well) in serum-free media containing FGF2 (10 ng/mL) and heparin (2 μg/mL) and were then seeded via automated liquid handler into 96-well plates. Since we did not have a positive reference compound, we used a pseudo-positive “2× control” by seeding some wells at 2000 cells/well (20 cells/μL) rather than 1000 cells/well (10 cells/μL). Each well contained 0.1% DMSO vehicle control or 0.1% DMSO + 1 μM of a single compound from the Ontario Institute for Cancer Research (OICR) tool compound library (TCL). The OICR TCL consists of 400 small molecule agonists and inhibitors, the majority of which are either clinical trial phase or approved therapeutics. RSPCs were incubated with molecules for 7 days in a monolayer culture and were then live-cell imaged to determine the number of Hoechst-positive nuclei and the total area of GFP expression in each well. Data were normalized as a percent of control and a hit was defined as a compound that resulted in an increase in both nuclei number and GFP area that were each 3 standard deviations (SD) above the control mean (Fig. 1, Table S1). Hits also were validated visually to ensure quantification was based on enumeration of Hoechst-stained nuclei and the area of GFP-expressing cells in the well and not due to compound precipitation or other artifacts (Figure S1), and the GFP area-to-nuclei number ratio was calculated to assess the compounds for cell hypertrophy effects (Table S1). Control conditions showed low variability and large signal-to-noise ratios that enabled hit identification with statistical confidence (Table S2). We performed two full library screens that independently resulted in 7 hits (screen 1) and 6 hits (screen 2) for a total of 12 unique hits identified (Fig. 1b–e; Table S1).

Only the synthetic glucocorticoid (GC) agonist prednisolone (Pred) met the hit criteria in both screens. Another synthetic GC agonist, Dex, was a hit in screen 2. Both Pred and Dex had GFP:nuclei ratios close to 1 (Table S1), indicating they did not cause cell enlargement relative to 0.1% DMSO control. The OICR TCL contains two other drugs classified as corticosteroids: hydrocortisone (HC) and prednisone. However, given that the activity of prednisolone is 4× that of HC and the activity of dexamethasone is 25× that of HC, it may not be surprising HC was not a hit at the same 1-μM concentration [30]. Likewise, prednisone is an inactive pro-drug/metabolite that requires conversion to prednisolone by 11β-hydroxysteroid dehydrogenase (11β-HSD) to be able to cross the cell membrane and have pharmacological effects [31]. Nonetheless, a hypergeometric statistical test still found that 2 hits out of 4 GC-class compounds in the library were a greater hit rate than would be expected by chance (Table S1). As GCs previously have been found to modulate neural progenitor proliferation and differentiation [32, 33], and are known to be important in retinal development and maturation [34], the synthetic GC agonists became our lead hits of interest.

Other hit compounds identified during the screening that target molecular signaling pathways known to regulate various stem and progenitor cell types included the Rho/ROCK inhibitor, Thiazovivin; the dipeptidyl peptidase IV inhibitor, MK-0431; the indoleamine dioxygenase inhibitor, INCB024360; the a2-adrenergic receptor agonist, Guanabenz; the cathepsin K inhibitor, Odanacatib; and the HDAC inhibitor, sodium butyrate (Table S1). However, some of those compounds did have GFP:nuclei ratios much greater than 1, indicating increased cell number and cell size vs control. A 5-point dose-response assay verified that Pred and Dex resulted in the greatest increase in RSPCs (2.32-fold and 1.96-fold of control, respectively) at the 1-μM dose (Fig. 1f). The compounds with the next highest effects were Thiazovivin (1.92-fold at 1 μM) and Guanabenz acetate (1.67-fold at 0.1 μM). Therefore, MTS successfully identified several putative hit compound classes, with synthetic GC agonists as the lead hit compound class.

### Glucocorticoid agonists stimulate mouse retinal stem and progenitor cell proliferation in vitro via both glucocorticoid receptor and mineralocorticoid receptor signaling

**Fig. 2:**
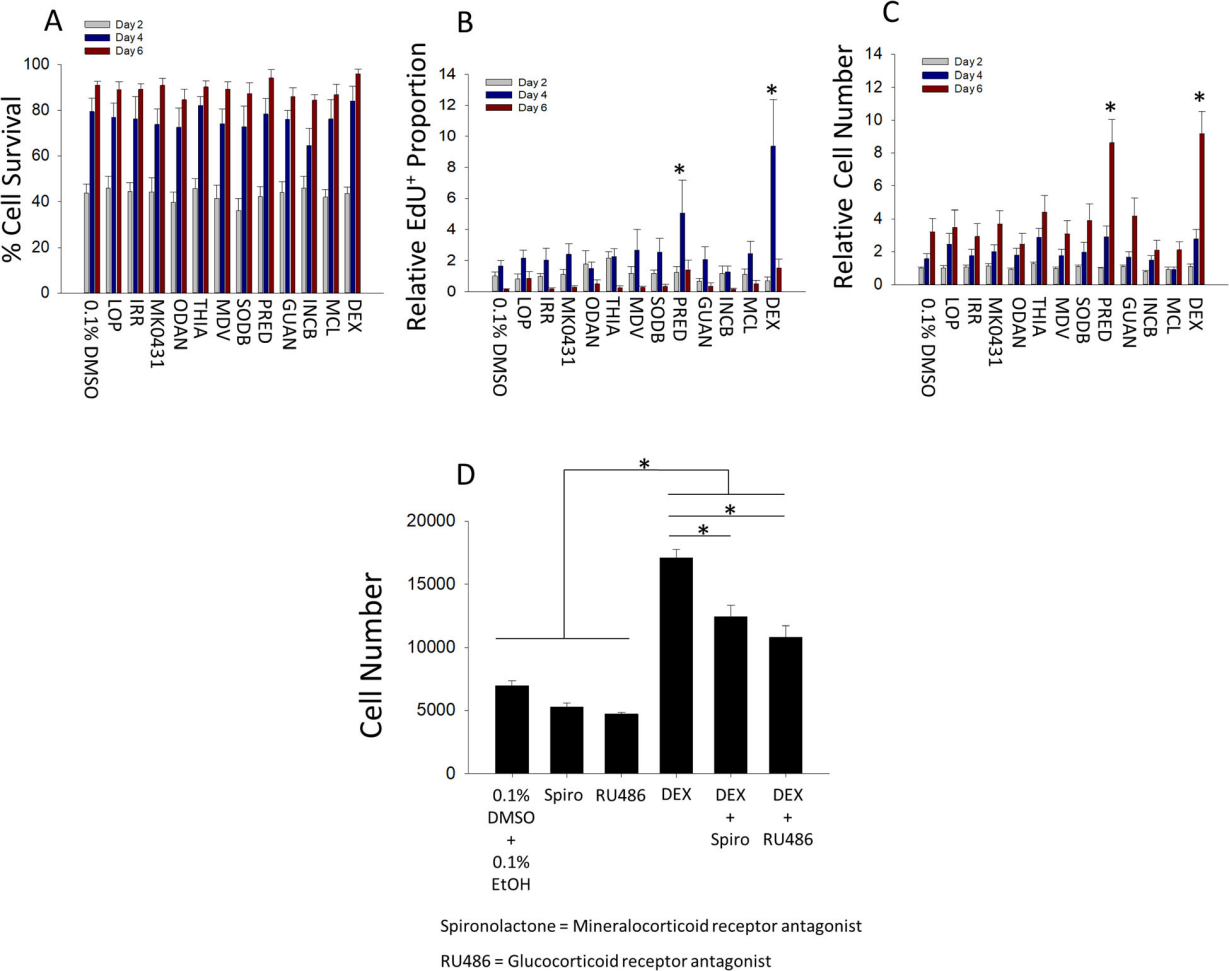
Glucocorticoid agonists increase retinal stem and progenitor cell proliferation through glucocorticoid receptor and mineralocorticoid receptor signaling in mice. **a**–**c** Quantification of growth parameters for RSPCs at day 2, day 4, and day 6 of a monolayer growth assay. *Significantly different from control within that time point. **a** The proportion of live cells was not significantly different across drug treatments (*p* = 0.55), and there was no interaction between drug and time (*p* = 0.99). There was a significant effect of time on the proportion of live cells, and all times were significantly different (two-way ANOVA *F* (_2, 195_) = 261.9, *p* < 0.001; Holm-Sidak post hoc test, *p < 0.05). *N* = 6 for all conditions at each time point. **b** The proportion of proliferating EdU-labeled cells relative to control. There was a significant interaction between drug and time (two-way ANOVA *F* (_24, 195_) = 2.59, *p* < 0.001; Holm-Sidak post hoc test, *p < 0.05). *N* = 6 for all conditions at each time point. **c** The total number of cells relative to control. There was a significant interaction between drug and time (two-way ANOVA *F* (_24, 429_) = 3.79, *p* < 0.001; Holm-Sidak post hoc test, *p < 0.05). *N* = 12 for all conditions at each time point. **d** Total RSPC number at the end of a 7-day monolayer growth assay with 0.1% DMSO and 0.1% EtOH in all conditions. Control was significantly different from all Dex treatment conditions but not spironolactone (Spiro) or RU486 alone (one-way ANOVA *F* (_5, 12_) = 67.88, *p* < 0.001; Holm-Sidak post hoc test, *p < 0.05). *N* = 3 for RU486 alone. *N* = 4 for all other groups. Data are mean ± SEM

Here, we investigated the cell biological effects that underpinned the ability of the synthetic GC agonists and other hit molecules to effect the increases in RSPC number detected via MTS. We dissociated primary RSC spheres to single-cell suspensions of RSPCs and then performed a 6-day monolayer cell culture assay, in laminin-coated 24-well plates (4 cells/μL, 2000 cells/well), to assess survival and proliferation of cells. We analyzed 3 time points during the assay: day 2, day 4, and day 6. To assess whether any hit compounds affected cell death, we compared the total cell number at each time point with the number of cells that were positive for the cell membrane-impermeable nucleic acid stain ethidium homodimer (EthD-1), which was pulsed into the culture 15 min prior to each time point (Figure S2A-B). None of the hit compounds had any effect on cell survival at any time point (Fig. 2a).

There was a remarkable amount of cell death at day 2, as only ~ 40%–45% of the nuclei remained unlabeled for EthD-1 in all conditions. However, the proportion of cell death decreased progressively as ~ 80% of the nuclei were unlabeled by EthD-1 at day 4 and that increased to ~ 90% or above by day 6. Such significant cell death in the first days of the assay likely explains why an increase in the total cell number was not observed until day 6 of the assay, when only the Pred and Dex conditions showed a significant increase compared to control (Fig. 2c). To assess whether any hit compounds affected cell proliferation, we compared the proportion of the total nuclei that had incorporated the thymidine analog 5-ethynyl-2′-deoxyuridine (EdU), which was pulsed for 3 h prior to fixation at each time point (Figure S2C-D). EdU is incorporated into the DNA of cells during the DNA synthesis phase (S phase) of the cell cycle and is subsequently labeled by an azide-containing fluorescent dye to enable detection of proliferating cells [35]. In the 0.1% DMSO control condition, EdU-labeling was highest at day 4 and was almost completely absent by day 6. Only Pred and Dex significantly increased the proportion of EdU-labeled cells compared to control. At day 4, Dex had a ~ 5.6-fold increase in EdU labeling while Pred had a ~ 3-fold increase. Thus, both synthetic GC agonists increased the maximum proportion of RSPCs in the S phase of the cell cycle at the peak of proliferation on day 4 and increased the total cell number at the end of the assay, whereas no other hit compound resulted in a significant increase in EdU labeling or cell number.

To further resolve the molecular signaling pathways mediating the proliferative effect of synthetic GC agonists on RSPCs, we investigated whether a chemical antagonist to mineralocorticoid receptor (MR) (spironolactone) or glucocorticoid receptor (GR) (RU486) could abolish the increased cell number induced by Dex treatment. We focused on Dex because it has a much higher potency than Pred (as mentioned above) and a longer duration of action (36–72 h for Dex vs 12–36 h for Pred) [30]. In mouse cells, Dex treatment increased cell number by 2.45-fold compared to control (Fig. 2d). When MR was blocked, the Dex effect was reduced to 1.78-fold of control, indicating that 46.2% of the increase in cell number was mediated through MR signaling. When GR was blocked, the Dex effect was reduced to 1.55-fold of control, indicating that 62.1% of the increase in cell number was mediated through GR signaling. This indicates that both MR and GR pathways are activated by Dex and contribute to its proliferative effect on mouse RSPCs. This contrasts with other neural progenitors, such as human hippocampal neural progenitors, for which MR signaling has been shown to have a proliferative effect, whereas GR signaling inhibits progenitor proliferation [32].

### Glucocorticoid agonism has differential effects on proliferation and self-renewal of adult stem and progenitor cells from different tissues

**Fig. 3:**
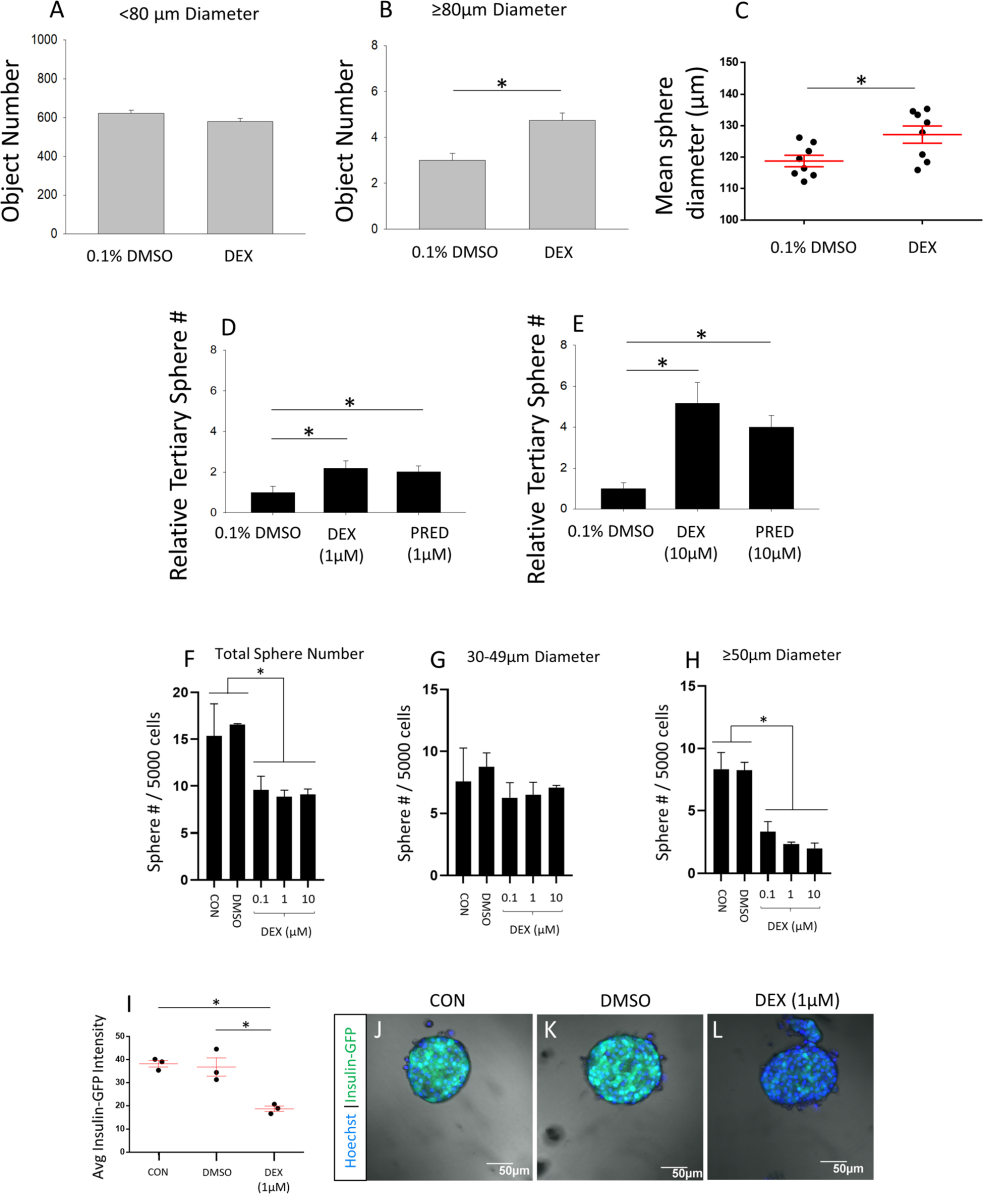
Dexamethasone increases retinal stem cell sphere size, number, and self-renewal but inhibits growth and insulin expression of pancreatic multipotent progenitor spheres. **a**–**c** Quantification of sphere diameter after a 7-day free-floating clonal sphere assay with actin-GFP mouse-derived secondary RSPCs in 0.1% DMSO vehicle or 1 μM Dex. **a** The number of cell colonies less than 80 μm in diameter was not significantly different between 0.1% DMSO vehicle control and 1 μM Dex treatment. *N* = 8 per condition. **b** Cell colonies greater than 80 μm in diameter increased in number by 1.63-fold with Dex treatment. (*t* test *t* (_14_) = − 5, *p* < .001; *N* = 8 per condition). **c** RSC sphere colonies 80 μm or above in diameter demonstrated an overall increase in size with Dex treatment (*t* test *t* (_14_) = 2.56, *p* < .05; *N* = 8 per condition). *Significantly different from 0.1% DMSO control. **d**, **e** Quantification of the number of tertiary RSC sphere colonies grown in drug-free media after prior exposure of secondary cells to the indicated compounds at 1 μM or 10 μM during a 7-day free-floating clonal sphere assay. Exposure to Dex and Pred increased the number of RSC spheres after passaging by 2.2-fold and 2-fold, respectively, at 1 μM (**d**; one-way ANOVA *F* (_2, 15_) = 4.02, *p* = 0.04; Fisher LSD post hoc test, *p < 0.05; *N* = 6 per group), and increased RSC sphere number by 5.2-fold and 4-fold, respectively, at 10 μM (**e**; one-way ANOVA *F* (_2, 6_) = 9.6, *p* = 0.014; Fisher LSD post hoc test, *p < 0.05; *N* = 3 per group). *Significantly different from 0.1% DMSO control. **f**–**h** Quantification of adult PMP spheres after a 7-day free-floating clonal sphere assay. **f** The total number of spheres ≥ 30 μm was significantly reduced by all concentrations of Dex tested (one-way ANOVA *F* (_4, 10_) = 4.76, *p* = 0.02; post hoc test, Holm-Sidak post hoc test, *p < 0.05; *N* = 3 experiments). **g** The number of spheres 30–49 μm in diameter was not influenced by Dex treatment at any concentration tested. *N* = 3 experiments. **h** The number of spheres ≥ 50 μm was significantly reduced by all concentrations of Dex tested (one-way ANOVA *F* (_4, 10_) = 16.12, *p* < 0.001; Holm-Sidak post hoc test, *p < 0.05; *N* = 3 experiments). *Significantly different from indicated conditions. **i**–**l** The intensity of MIP-GFP expression in PMP spheres. **i** Quantification of the intensity of MIP-GFP expression in PMP spheres (one-way ANOVA *F* (_2, 6_) = 18.35, *p* = 0.0028; Holm-Sidak post hoc test, *p < 0.05; *N* = 3 replicates per condition). **j**–**l** Representative confocal projection images of MIP-GFP expression in PMP spheres. Data are mean ± SEM

To further characterize the differential effects of GC signaling on different adult stem cell populations, we treated adult stem cells from two different germ layers with Dex during 7-day clonal sphere-forming assays—RSCs and pancreatic multipotent precursor cells (PMPs). For RSC spheres, a threshold of 80 μm in diameter is used to distinguish between spheres that arise from an RSC (≥ 80 μm) and those that arise from a progenitor cell (< 80 μm), as determined by the diminished passaging ability of spheres below 80 μm in our hands. Dex treatment increased the total number of RSC spheres greater than 80 μm in diameter (Fig. 3a, b) and increased the maximum diameter of these RSC spheres (Fig. 3c).

Thus, Dex likely increased progenitor proliferation, which increased the sphere size. The increase in sphere number in response to Dex may be due to enhanced progenitor proliferation, resulting in more spheres exceeding 80 μm in diameter, or could be due to a subpopulation of quiescent RSCs that normally remain dormant in a culture becoming stimulated to proliferate and form spheres. To assess if synthetic GC agonists influence RSC self-renewal, spheres ≥ 80 μm in diameter that were initially grown in 0.1% DMSO, Dex, or Pred were passaged into single-cell suspensions of tertiary cells and re-plated for a subsequent clonal sphere-forming assay in serum-free media + FGF2 and heparin (with no compounds or DMSO present). Both Dex- and Pred-treated spheres resulted in similar increases in the number of tertiary spheres compared to the 0.1% DMSO control (~ 2-fold increase at 1 μM and over 4-fold increase at 10 μM), indicating GC agonism can stimulate symmetric RSC self-renewal and expansion (Fig. 3d, e). Therefore, it appears the synthetic GC agonists increase retinal progenitor proliferation and RSC self-renewal.

For PMPs spheres, ≥ 30 μm in diameter was set as the threshold for quantification, as they are typically smaller than RSC spheres, and objects less than 30 μm in diameter tend to form due to aggregation given the higher seeding density (20 cells/μL vs 10 cells/μL). Another important difference is that PMP spheres have very low passage efficiency and rarely form secondary spheres [28]. In contrast to RSC spheres, PMP sphere growth was suppressed by Dex treatment. The total number of spheres was significantly reduced, which could be due to fewer sphere colonies reaching the 30-μm threshold resultant of attenuated proliferation, or alternatively, some sphere-initiating pancreatic progenitors may remain quiescent in culture in response to GC agonism (Fig. 3f). However, while the number of spheres between 30 and 49 μm in diameter did not change (Fig. 3g), there was a significant reduction in spheres 50 μm or above in diameter, indicating GC agonism likely inhibits pancreatic progenitor proliferation rather than sphere initiation (Fig. 3h). This contrasting outcome to that observed for RSC spheres demonstrates that adult stem and progenitor cells from different tissues are regulated by GC signaling in a cell type-specific manner. Next, using a mouse insulin promoter (MIP)-GFP mouse line, we examined whether Dex treatment would influence the early fate specification of PMPs. PMPs themselves are known to express insulin and the MIP-GFP reporter at a low level, whereas their differentiated beta cell progeny expresses high levels of insulin and the MIP-GFP reporter [36, 37]. During sphere growth, an increase in reporter expression relative to control would denote the differentiation of PMPs toward beta cells, whereas a relative decrease in reporter expression would denote fate specification toward non-insulin-expressing pancreatic progeny. Treatment of PMPs with Dex during 7 days of sphere growth resulted in decreased MIP-GFP reporter expression relative to control, indicating that GC agonism likely has a differentiation effect, directing PMPs toward non-beta cell progeny (Fig. 3i–l). Furthermore, a differentiation effect could explain the decrease in PMP proliferation caused by Dex. Thus, we have identified that GC signaling also can regulate adult pancreatic precursor cell differentiation and proliferation, but with different effects compared to retinal stem and progenitor cells.

### Glucocorticoid agonism does not change the differentiation profile of retinal progenitor cells

**Fig. 4:**
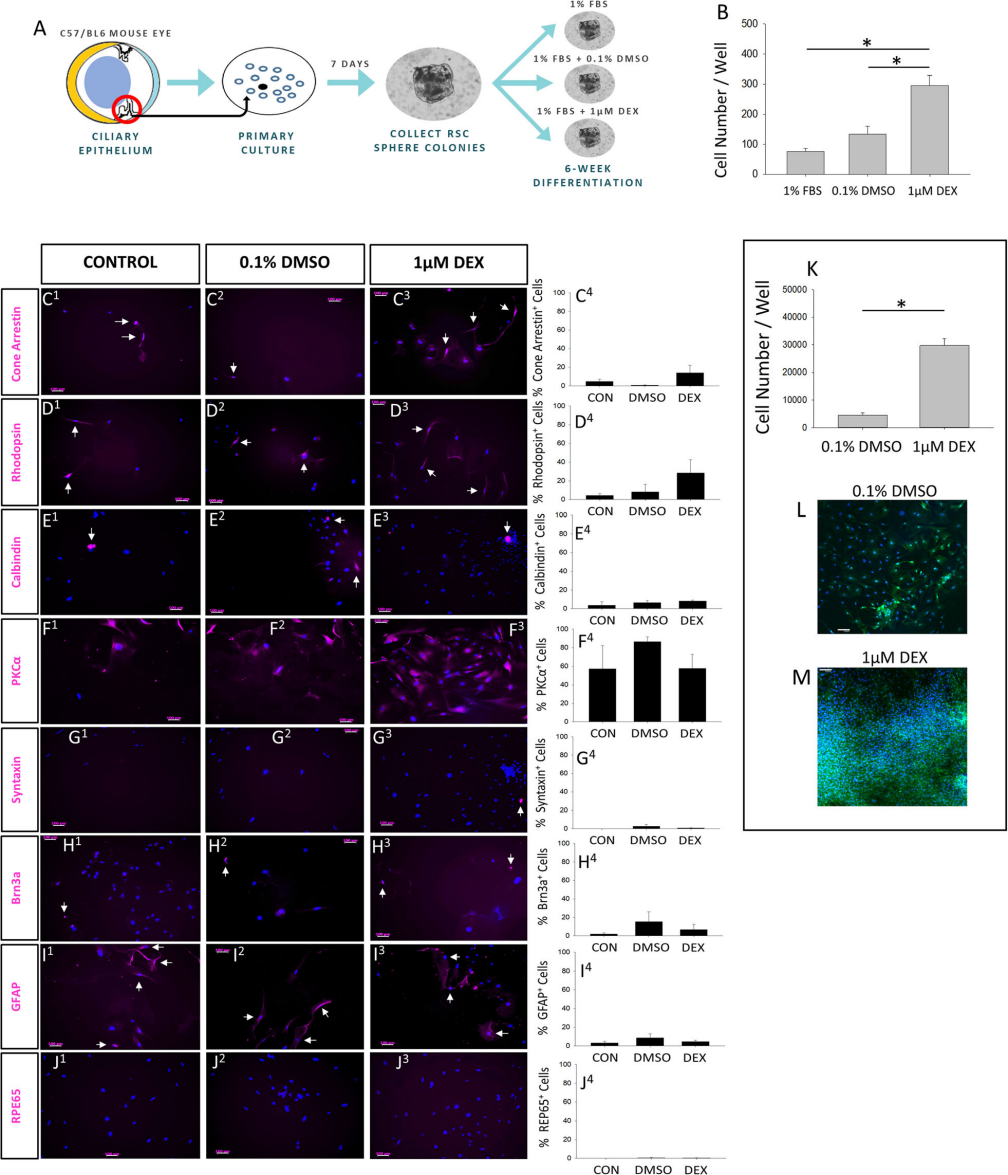
Dexamethasone does not affect the differentiation profile of retinal progenitor cells. **a** Schematic overview of the RSPC differentiation assay. **b** Quantification of cell number per well after 6 weeks in the indicated differentiation conditions (one-way ANOVA *F* (_2, 8_) = 16.46, *p* = 0.001; Holm-Sidak post hoc test, *p < 0.05; *N* = 3–4 per group)**.** *Significantly different between indicated conditions. **c**–**j** IHC images and quantification of mature retinal cell type markers following 6 weeks of differentiation across the indicated conditions. *N* = 3–4 per group. The nuclei labeled with Hoechst stain (blue). White arrows indicate cells positive for cell type markers. **k**–**m** Images and quantification of secondary RSPCs from actin-GFP mice grown as monolayers for 6 weeks in 1% FBS differentiation media (*t* test *t* (_14_) = − 9.54, *p* < .001; *N* = 8 wells per condition). *Significantly different between indicated conditions. Data are mean ± SEM

To investigate whether GC signaling influences the differentiation profile of RSC progeny, we added 1 μM Dex for the entire duration of a 6-week differentiation protocol, where whole clonal RSC spheres derived from C57/BL6J mice were plated in laminin-coated 24-well plates (Fig. 4a). We used these mice due to the potential for retinal cell defects caused by mutations in albino mouse strains and other C57 strains [38]. The proliferative effect of Dex was evident as the Dex-treated wells had an average of 2.3-fold increase in cell number compared to the 0.1% DMSO vehicle at the end of the 6-week differentiation (Fig. 4b). Markers for all differentiated retinal cell types were assessed, which included cones (cone arrestin), rods (rhodopsin), horizontal cells (calbindin), bipolar cells (PKCα), amacrine cells (syntaxin), retinal ganglion cells (Brn3a), Müller glia (GFAP), and retinal pigmented epithelium (RPE65). However, no difference in the proportion of any cell type was detected across all conditions (Fig. 4c–j). On average, bipolar cells appeared to be the most frequent cell type produced at ~ 57–84% of progeny across groups, whereas RPE cells were nearly undetected ranging from 0 to 0.46% of progeny. Across the 3 treatment conditions, rods were detected at an average range of 4–28%, cones ranged from 0.8 to 14%, and RGCs were 2–15%. Similar to some previous reports using 2D culture [13, 19, 22, 23], RSC progeny did not take on morphological features of mature retinal cell types, such as photoreceptor outer segments. However, this appears to be dependent on culture conditions, as retinal progenitors more readily acquire mature morphology in 3D/co-cultures cultures [39, 40] and RSC progeny develop mature morphology after transplantation into the retina [14, 24]. Despite the range of cell type proportions observed between conditions, cell type output was highly variable across biological replicates within conditions, and thus, there were no statistically significant differences in cell type proportions between the treatment groups. Nonetheless, due to the 3.58-fold Dex-mediated increase in cell number compared to the 1% FBS condition, the absolute number of cell types produced (such as photoreceptors) is greater with Dex treatment. In that regard, it is notable that, in a separate 6-week assay where RSPCs were seeded as a monolayer instead of plating whole spheres, the proliferative effect of Dex was even greater (6.67-fold increase in cell number vs 0.1% DMSO control; Fig. 4k–m). However, that experiment also used actin-GFP mice, not C57/BL6J mice, so both assay format and strain-specific differences may influence the degree of GC-mediated proliferation of RSPCs. In sum, GC agonism resulted in a pronounced increase in RSPC proliferation but did not influence cell type specification during retinal progenitor differentiation.

### Glucocorticoid agonism in vivo induces proliferation in the ciliary epithelium of the mouse eye but does not expand the retinal stem cell population

**Fig. 5:**
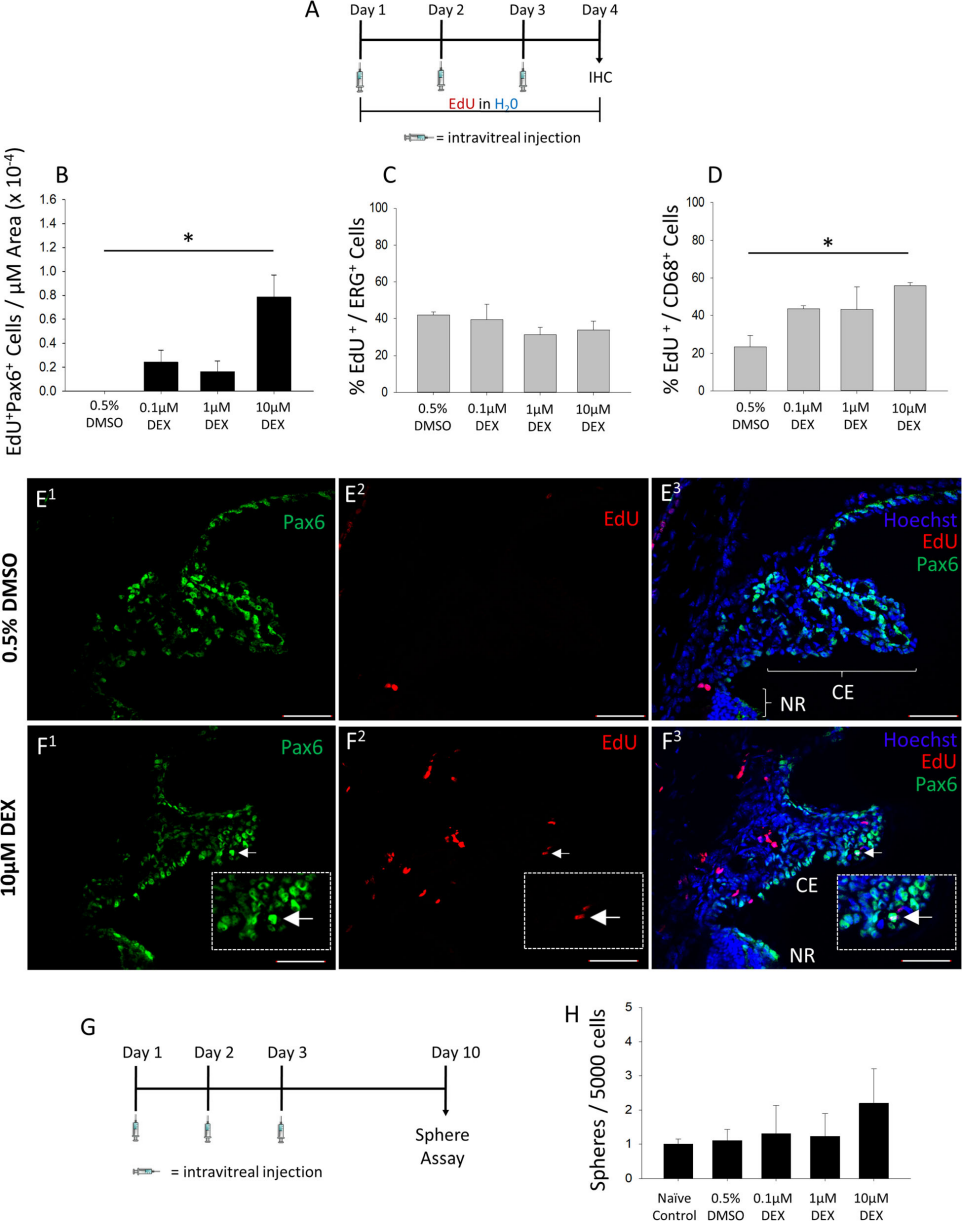
Intravitreal dexamethasone injection induces ciliary epithelium proliferation but does not expand the retinal stem cell population in vivo*.* **a** Schematic of the intravitreal injection paradigm followed by endpoint IHC. **b** Quantification of Pax6+EdU co-labeled cells relative to the total CE area in the eyes treated with 0.5% DMSO vehicle or indicated Dex concentrations (one-way ANOVA *F* (_3, 15_) = 6.21, *p* = 0.006; Holm-Sidak post hoc test, *p < 0.05; *N* = 3–6 eyes per condition). *Significantly different between indicated conditions. **c** The proportion of ERG + EdU-co-labeled endothelial cells relative to the total number of EdU-labeled cells in the ciliary body for each indicated condition. *N* = 3–6 eyes per condition. **d** The proportion of CD68 + EdU-co-labeled microglia/macrophages relative to the total number of EdU-labeled cells in the ciliary body for each indicated condition (one-way ANOVA *F* (_3, 14_) = 6.89, *p* = 0.004; Fisher Holm-Sidak post hoc test, *p < 0.05; *N* = 3–6 eyes per condition). *Significantly different between indicated conditions. **e**, **f** Representative images of Pax6 IHC and EdU labeling in the ciliary body and peripheral retina of mouse eyes exposed to 0.5% DMSO vehicle or indicated Dex concentrations. The nuclei labeled with Hoechst stain. White arrows indicate Pax6 + EdU co-labeled cells; 10-μm-thick sections; 50-μm scale bars. **g** Schematic of intravitreal injection paradigm followed by endpoint primary sphere-forming assay. **h** Quantification of RSC sphere frequency relative to naive un-injected control after a 7-day clonal sphere growth assay following intravitreal injection of indicated conditions. *N* = 6 eyes per group. Data are mean ± SEM

GCs, including dexamethasone, are commonly used clinically for anti-inflammatory therapy in the eye [34, 41]. Yet, despite this widespread use, whether GC agonism stimulates adult RSC or CE proliferation has not been examined to our knowledge. Here, we investigated whether in vivo delivery of Dex can overcome the inhibitory signals in the RSC niche and induce proliferation in the adult mouse eye. We used an intravitreal injection paradigm where each eye received one injection per day for 3 days and was then collected and fixed 24 h after the final injection for subsequent immunohistochemical (IHC) analyses (Fig. 5a). We delivered 3 different concentrations of Dex (to achieve final concentrations of 0.1 μM, 1 μM, or 10 μM in vivo) or 0.5% DMSO as a vehicle control. For the whole duration of the experiment, the thymidine analog EdU was delivered via the drinking water so it would be incorporated by, and label, any cell that entered the S phase of the cell cycle. There are currently no exclusive markers for RSCs. Pax6, which is a marker of retinal progenitor cells during development, has been shown to be highly expressed and functionally required in RSCs [42]. Pax6 is also known to label amacrine cells and both layers of the ciliary epithelium in the adult mouse eye [43, 44]. However, the amacrine cells and CE cells marked by Pax6 are easily distinguished based on anatomical location in the retina vs the CE. Therefore, we quantified the proportion of Pax6-stained cells in the CE that were co-labeled with EdU to determine the level of CE proliferation in vivo, which may be an indication of RSC proliferation. The 10-μM dose of Dex resulted in a significant increase in Pax6-positive CE cells co-labeled with EdU (Fig. 5b, e, f; Figure S3), indicating GC agonism can induce CE proliferation in vivo and, thus, potentially stimulate RSCs as well. It also was evident that there were Pax6-negative cells that were labeled with EdU in the ciliary body. To determine what are the non-CE cell types labeled by EdU, we co-stained for the endothelial cell nuclear marker, ERG, and activated microglia/macrophage marker, CD68. There was a consistent proportion of ERG + EdU-co-labeled cells across all conditions, indicating there is a basal population of proliferating endothelial cells labeled by EdU that is not enhanced by GC agonism (Fig. 5c; Figure S4A-B). In contrast, the 10-μM dose of Dex increased the proportion of CD68 + EdU co-labeled cells compared to the 0.5% DMSO control (Fig. 5d; Figure S4C-D). This result could be an indication of microglia/macrophage EdU incorporation due to GC-mediated cell death/DNA repair activity [45]. However, no evidence of co-localization of activated caspase 3 with CD68 or EdU was found (data not shown).

To investigate whether in vivo GC agonism not only causes CE proliferation but increases the number of RSCs in the CE, we performed a similar intravitreal injection paradigm, but this time waited 7 days following the final injection and then dissected the primary ciliary epithelium to perform 7-day clonal sphere assays (Fig. 5g). In vitro, single RSCs proliferate to form clonal spheres of cells. Thus, the number of RSC spheres is proportional to the number of endogenous RSCs and can be used to measure changes in the number of RSCs in the CE (Balenci and van der Kooy [46]; Tropepe et al. [11]). There were no significant differences in the number of spheres produced across treatment conditions (Fig. 5h). Thus, whereas GC agonism in vivo does induce CE cells to proliferate, which may include stimulation of RSC proliferation, it does not result in increased RSC symmetric self-renewal and expansion. Therefore, if RSCs are being stimulated to exit quiescence and proliferate, they are most likely proliferating asymmetrically.

## Discussion

In this study, we developed an MTS screening method to identify small molecules that expand the number of retinal stem and progenitor cells in culture. We demonstrated that our lead hit molecules, synthetic glucocorticoid agonists, enhance RSPC proliferation and RSC self-renewal in vitro, and have the capacity to induce CE proliferation in vivo. Synthetic glucocorticoids signal through the glucocorticoid and mineralocorticoid receptors and also are regulated at the pre-receptor level by the isozymes 11-β-HSD1 and 11-β-HSD2 [47, 48]. The GR, MR, and the 11β-HSD1 and 11β-HSD2 isozymes are known to be expressed in the CE of humans and rodents [47]. In a recent study, our lab performed RNAseq on primary RSC spheres derived from two different strains of mice [49]. The transcriptomic dataset shows that mouse RSC-derived clonal spheres express the GR gene (Nr3c1), the MR gene (Nr3c2), and 11β-HSD isozymes 1 and 2 (hsd11b1, hsd11b2), with Nr3c1 showing particularly high expression (Figure S5). Given that the expression level of GR protein determines the magnitude of glucocorticoid response, these data support the findings of this study that RSPC cultures are responsive to GR agonists dexamethasone and prednisolone [48]. By inhibiting GR or MR signaling, we found that both pathways act in mouse RSPCs to produce the observed increase in cell number caused by dexamethasone treatment. However, it remains to be determined if the cell biological effects mediated by each receptor result from their direct signaling mechanisms or crosstalk via downstream factors such as co-activator regulation, chromatin landscape changes, posttranslational modifications, or interactions with other signaling pathways [48].

Another factor that could influence the outcome of GR and MR agonism is the dose. Our screen was designed to identify compounds that increased the number of RSPCs in the culture at a 1-μM concentration. The highest dose tested in this study in vitro was 10 μM: cell expansion was tested with a dose-response assay, and self-renewal was tested with a sphere passaging/self-renewal assay. In both cases, 10 μM Dex and Pred produced significant increases. Compared to 1 μM, 10 μM Dex and Pred had slightly lesser effects on cell number but greater effects on self-renewal. Generally, GCs are thought to decrease neurogenesis and proliferation of neural stem and progenitor cells [50]. However, an in vitro study on human hippocampal progenitors by Anacker et al. (2013) found that low-dose cortisol (100 nM) operated through MR to enhance progenitor proliferation, whereas high-dose cortisol (100 μM) acted via GR to inhibit progenitor proliferation. That observation is in line with the higher affinity of cortisol for MR than GR [51]. Also notable, at both high and low doses of cortisol, they found a reduction in neurogenesis and differential influence on astrogliogenesis. This contrasts with our finding that Dex had no effect on retinal progenitor differentiation and output of any specific retinal cell types. Yet, in our study, only 1 μM Dex was tested during differentiation, so the possibility remains that there could be a dose-dependent effect of GR agonism on retinal progenitor differentiation. However, since here we are discussing progenitors of different species and different tissues, and glucocorticoids are well-known to have cell type-specific effects, these differences may occur regardless of dose [48, 52]. Indeed, our experiments with mouse PMPs resulted in findings that directly contrast our RSPC results. Dex treatment mediated a reduction in clonal PMP sphere size and number, as well as decreased insulin expression, which is indicative of decreased proliferation and altered differentiation [36, 37]. These results were achieved at the same 1 μM and 10 μM Dex doses that had no effect on retinal progenitor differentiation and increased RSPC proliferation confirming cell-specific differences in the effect of glucocorticoids on adult progenitor cells from different tissues.

It has been demonstrated previously that drugs which increase stem cell proliferation and self-renewal in vitro can have regenerative effects when applied in vivo [53–55]. We hypothesized this may also be true for RSCs, and therefore, we tested several concentrations of Dex in vivo in the mouse eye via a series of intravitreal injections. We found that a 10-μM dose of Dex induced a significant increase in EdU labeling in the CE, indicative of CE proliferation. Since we used Pax6 to label the CE, and Pax6 is known to be expressed in retinal progenitors and RSCs, it is possible that proliferating RSCs and RSC-derived progenitors were the cells being labeled by EdU in the CE [42]. Further, while the frequency of Pax6+EdU co-labeling was very low, that is concordant with previous reports of RSCs being very rare at ~ 1 in 500 CE cells [13]. Likewise, rarity and quiescence are the common features of other adult stem cells, such as hematopoietic and neural stem cells [6, 56]. However, without a distinctive molecular marker for RSCs or newborn progenitors, this interpretation cannot be determined directly via immunohistochemistry.

We also found that Dex increased the proportion of CD68 + EdU-co-labeled cells in the CE and did not find any evidence this was due to DNA repair activity. This finding could be due to increased immune cell infiltration as, although GCs are well-known for their anti-inflammatory properties, recent studies have demonstrated that GCs can also mediate pro-inflammatory responses [34, 57]. Alternatively, GCs have been shown to increase microglia proliferation in the CNS in vivo [58]. Furthermore, Dex-mediated immunomodulation in the zebrafish eye can both delay or accelerate neuronal regeneration by MG cells depending on whether it is delivered pre- or post-injury [59]. Thus, it is possible that Dex may mediate CE proliferation (and potentially RSC activation) indirectly via immune modulation.

We sought to ascertain if there was an in vivo RSC self-renewal effect of Dex by performing the same intravitreal injection paradigm with a follow-up primary sphere assay 7 days after the injection period. However, no difference in sphere number resulted from Dex treatment, indicating Dex did not cause in vivo symmetric self-renewal and expansion of RSCs. Given that Dex does increase RSC proliferation in vitro and results in increased EdU labeling in the CE, Dex may be inducing asymmetric RSC division in vivo. This is in line with previously reported findings of exclusive asymmetric division of proliferating CE/RSCs in vivo [60, 61]. Furthermore, an asymmetric division is known to be the exclusive mode of division for several other adult stem and progenitor cells in vivo [6]. Alternatively, some studies have suggested that CE cell proliferation results from a general propensity of CE cells to reprogram/de-differentiate and become proliferative, rather than the division of rare, quiescent CE-RSCs [23, 62]. However, as mentioned previously, evidence that sphere colony-forming RSCs can be prospectively enriched via FACS supports, the interpretation that a pre-existing subset of CE cells possess proliferative competency and differentiation capacity [15, 42]. Further, more widespread CE proliferation and higher sphere colony formation frequency would be expected if all CE cells have cell fate plasticity and proliferative competency. Yet, until specific markers of RSCs are elucidated, it is inconclusive whether the GR agonist-mediated proliferation in the CE demonstrated herein is due to the direct stimulation of RSCs. It is also possible that a higher dose or more prolonged exposure could lead to RSC expansion in vivo.

Many stem cell signaling pathways have been demonstrated to regulate RSPC proliferation and RSC self-renewal. For instance, Wnt activation and Notch activation have each been shown to increase RSPC proliferation and symmetric self-renewal of RSCs (Balenci and van der Kooy [46]; Inoue et al. [63]). Also, Hedgehog signaling blockade has been shown to decrease the proliferation of RSPCs in culture [49], and mice with a Ptc^+/−^ mutation have an extended period of postnatal retinal progenitor proliferation in vivo [64]. However, since the discovery of RSCs onward, it has been postulated that the in vivo quiescence of RSCs in the adult mammalian eye is mediated by inhibitory factors in the RSC niche that impede the ability of exogenous factors to stimulate endogenous RSCs [11, 12, 27]. Notably, Balenci et al. [26] reported that lens and cornea-secreted BMP and sFRP proteins might be responsible for the quiescence of RSCs in vivo based on their ability to reversibly suppress RSC sphere growth in vitro*.* Coincidentally, glucocorticoid signaling has been shown to regulate several molecular signaling pathways, including Wnt signaling, Notch signaling, BMP signaling, and Hedgehog signaling in various progenitor populations and tissues, including neural progenitors [32]. Thus, it will be important to investigate the effect of glucocorticoid signaling on the regulation of these canonical stem cell signaling pathways in retinal stem and progenitor cells to determine if modulation of these pathways explains the proliferative effect of Dex on the CE in vivo. It also may be possible that concurrent blockade of BMP and/or sFRP proteins will enhance the proliferative effect of Dex in vivo and lead to a greater therapeutic potential for endogenous retinal repair.

## Conclusions

In summary, this study used compound screening to reveal that the glucocorticoid and mineralocorticoid signaling pathways regulate retinal stem cell self-renewal and proliferation. However, the synthetic glucocorticoid agonist dexamethasone does not influence RSPC cell fate determination in vitro. Furthermore, injection of dexamethasone in the adult mouse eye stimulates proliferation of the ciliary epithelium, which may indicate activation of endogenous RSCs. As synthetic glucocorticoid agonists are commonly used clinically for the treatment of ocular diseases [52], this study raises the possibility that these drugs, which are already known to be safe in humans for ocular use, could be adapted for retinal regenerative therapy. And, more speculatively, it may be that RSC-mediated retinal regeneration is an as-of-yet unexamined outcome of ocular glucocorticoid administration in humans.

## Supplementary Information

**Additional file 1: Figure S1.** Visual confirmation that glucocorticoid agonists enhanced retinal stem and progenitor yield and was not due to artifacts. Images from the Celigo imaging cytometer showing 96-well plate wells at the end of a 7-day growth assay. Wells were treated with the indicated glucocorticoid agonist compounds. The nuclear channel, the GFP channel and merge demonstrate the ability to differentiate Hoechst and actin-GFP double-positive objects from debris and other artifacts that appear only in the nuclear channel. Visually, it is apparent dexamethasone and prednisolone have greater signal than the other conditions. Red arrows indicate artifacts that fluoresce in the blue nuclear channel that do not fluoresce in the GFP channel. 4x magnification. **Figure S2.** Representative staining of cell death marker ethidium homodimer (EthD-1) and thymidine analog EdU. (A-B) EthD-1 labeling at Day 2 in cells treated with (A) 0.1% DMSO, or (B) 1μM Dexamethasone. (C-D) EdU labeling at Day 4 in cells treated with (A) 0.1% DMSO, or (B) 1μM Dexamethasone. **Figure S3.** Intravitreal dexamethasone injection induces ciliary epithelium proliferation. (A-B) Representative images of Pax6 IHC and EdU labeling in the ciliary body of mouse eyes exposed to (A) 0.5% DMSO vehicle, or (B) 10μM Dexamethasone. Nuclei are labeled via Hoechst staining. White arrows indicate Pax6 + EdU co-labeled cells. Dashed line indicates inset. 10 μm-thick sections. **Figure S4.** EdU-positive cells co-label with endothelial and microglia/macrophage markers. (A-B) Representative images of ERG IHC and EdU labeling in the ciliary body of mouse eyes exposed to (A) 0.5% DMSO vehicle, or (B) 10μM Dexamethasone. (C-D) Representative images of CD68 IHC and EdU labeling in the ciliary body of mouse eyes exposed to (C) 0.5% DMSO vehicle, or (D) 10μM Dexamethasone. Nuclei are labeled via Hoechst staining. White arrows indicate co-labeled cells. 10 μm-thick sections. **Figure S5.** Glucocorticoid receptor, Mineralocorticoid receptor, and 11-β-HSD1 & 2 RNA expression in RSC spheres. Transcriptomic data showing the expression of the glucocorticoid receptor (Nr3c1), mineralocorticoid receptor (Nr3c2) and the two 11-β-HSD isozymes in RSC spheres, supporting the finding of retinal precursor sensitivity to GR agonists. This graph was created from RNAseq data collected in Khalili et al. 2018. Two different mouse strains were used to generate RSC spheres that were lysed and high-quality total RNA (RIN: 9–10) was subjected to directional RNA-sequencing library construction from three independent biological replicates per mouse strain. Sequencing was performed using GAIIx (Illumina, Inc., San Diego, CA; www.illumina.com). **Table S1.** Compounds that met hit criteria in at least one of two screens. **Table S2.** Screening quality metrics.

**Additional file 2:** Ontario Institute for Cancer Research (OICR) Tool Compound Library.

**Additional file 3:** Screening Plate Maps.

## Data Availability

The data that support the findings of this study are available from the corresponding author upon reasonable request.

## Abbreviations

CB: Ciliary body
CE: Ciliary epithelium
CMZ: Ciliary marginal zone
DEX: Dexamethasone
DMSO: Dimethylsulfoxide
EdU: 5-Ethynyl-2′-deoxyuridine
EthD-1: Ethidium homodimer
EtOH: Ethanol
FBS: Fetal bovine serum
FGF: Fibroblast growth factor
FH: FGF+heparin
GC: Glucocorticoid
GR: Glucocorticoid receptor
HC: Hydroxycortisone
HDAC: Histone deacetylase
HSD: Hydroxysteroid dehydrogenase
ICC: Immunocytochemistry
IHC: Immunohistochemistry
MC: Mineralocorticoid
MG: Muller glia
MR: Mineralocorticoid receptor
MTS: Medium-throughput screening
NC: Naive control
NR: Neural retina
OICR: Ontario Institute for Cancer Research
PMP: Pancreatic multipotent progenitor
PRED: Prednisolone
RPC: Retinal progenitor cell
RPE: Retinal pigmented epithelium
RSC: Retinal stem cell
RSPC: Retinal stem/progenitor cell
SFM: Serum-free media
TCL: Tool compound library
