# Supplementary material for: Glucocorticoid agonists enhance retinal stem cell self-renewal and proliferation
Source: Stem Cell Res Ther. 2021 Jan 25;12:83. doi: 10.1186/s13287-021-02136-9 (PMC7831262; doi:10.1186/s13287-021-02136-9)
Supplement: Supplementary file 1 — Additional file 1: Figure S1. Visual confirmation that glucocorticoid agonists enhanced retinal stem and progenitor yield and was not due to artifacts. Images from the Celigo imaging cytometer showing 96-well plate wells at the end of a 7-day growth assay. Wells were treated with the indicated glucocorticoid agonist compounds. The nuclear channel, the GFP channel and merge demonstrate the ability to differentiate Hoechst and actin-GFP double-positive objects from debris and other artifacts that appear only in the nuclear channel. Visually, it is apparent dexamethasone and prednisolone have greater signal than the other conditions. Red arrows indicate artifacts that fluoresce in the blue nuclear channel that do not fluoresce in the GFP channel. 4x magnification. Figure S2. Representative staining of cell death marker ethidium homodimer (EthD-1) and thymidine analog EdU. (A-B) EthD-1 labeling at Day 2 in cells treated with (A) 0.1% DMSO, or (B) 1μM Dexamethasone. (C-D) EdU labeling at Day 4 in cells treated with (A) 0.1% DMSO, or (B) 1μM Dexamethasone. Figure S3. Intravitreal dexamethasone injection induces ciliary epithelium proliferation. (A-B) Representative images of Pax6 IHC and EdU labeling in the ciliary body of mouse eyes exposed to (A) 0.5% DMSO vehicle, or (B) 10μM Dexamethasone. Nuclei are labeled via Hoechst staining. White arrows indicate Pax6 + EdU co-labeled cells. Dashed line indicates inset. 10 μm-thick sections. Figure S4. EdU-positive cells co-label with endothelial and microglia/macrophage markers. (A-B) Representative images of ERG IHC and EdU labeling in the ciliary body of mouse eyes exposed to (A) 0.5% DMSO vehicle, or (B) 10μM Dexamethasone. (C-D) Representative images of CD68 IHC and EdU labeling in the ciliary body of mouse eyes exposed to (C) 0.5% DMSO vehicle, or (D) 10μM Dexamethasone. Nuclei are labeled via Hoechst staining. White arrows indicate co-labeled cells. 10 μm-thick sections. Figure S5. Glucocorticoid receptor, Mineralocorticoi [file 13287_2021_2136_MOESM1_ESM.pdf]

SUPPLEMENTARY INFORMATION

Glucocorticoid agonists enhance retinal stem cell self-renewal and proliferation

Kenneth N. Grisé, Nelson X. Bautista, Krystal Jacques, Brenda L.K. Coles, Derek van der Kooy

SUPPLEMENTARY FIGURES

Figure S1., Figure S2., Figure S3., Figure S4., Figure S5.

SUPPLEMENTARY TABLES

Table S1., Table S2.

SUPPLEMENTARY FIGURES

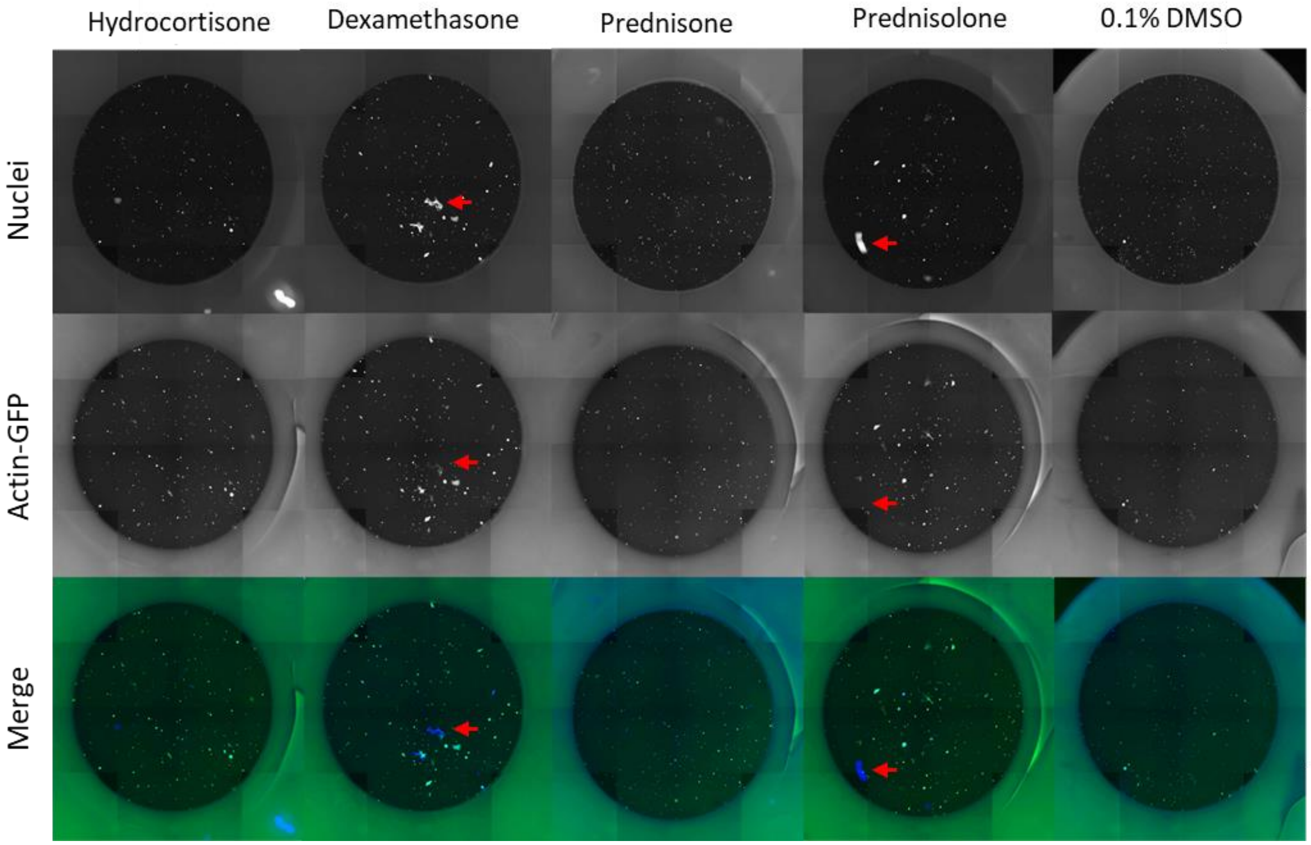

Figure S1. Visual confirmation that glucocorticoid agonists enhanced retinal stem and progenitor yield and was not due to artifacts

Images from the Celigo imaging cytometer showing 96-well plate wells at the end of a 7-day growth assay. Wells were treated with the indicated glucocorticoid agonist compounds. The nuclear channel, the GFP channel and merge demonstrate the ability to differentiate Hoechst and actin-GFP double-positive objects from debris and other artifacts that appear only in the nuclear channel. Visually, it is apparent dexamethasone and prednisolone have greater signal than the other conditions. Red arrows indicate artifacts that fluoresce in the blue nuclear channel that do not fluoresce in the GFP channel. 4x magnification.

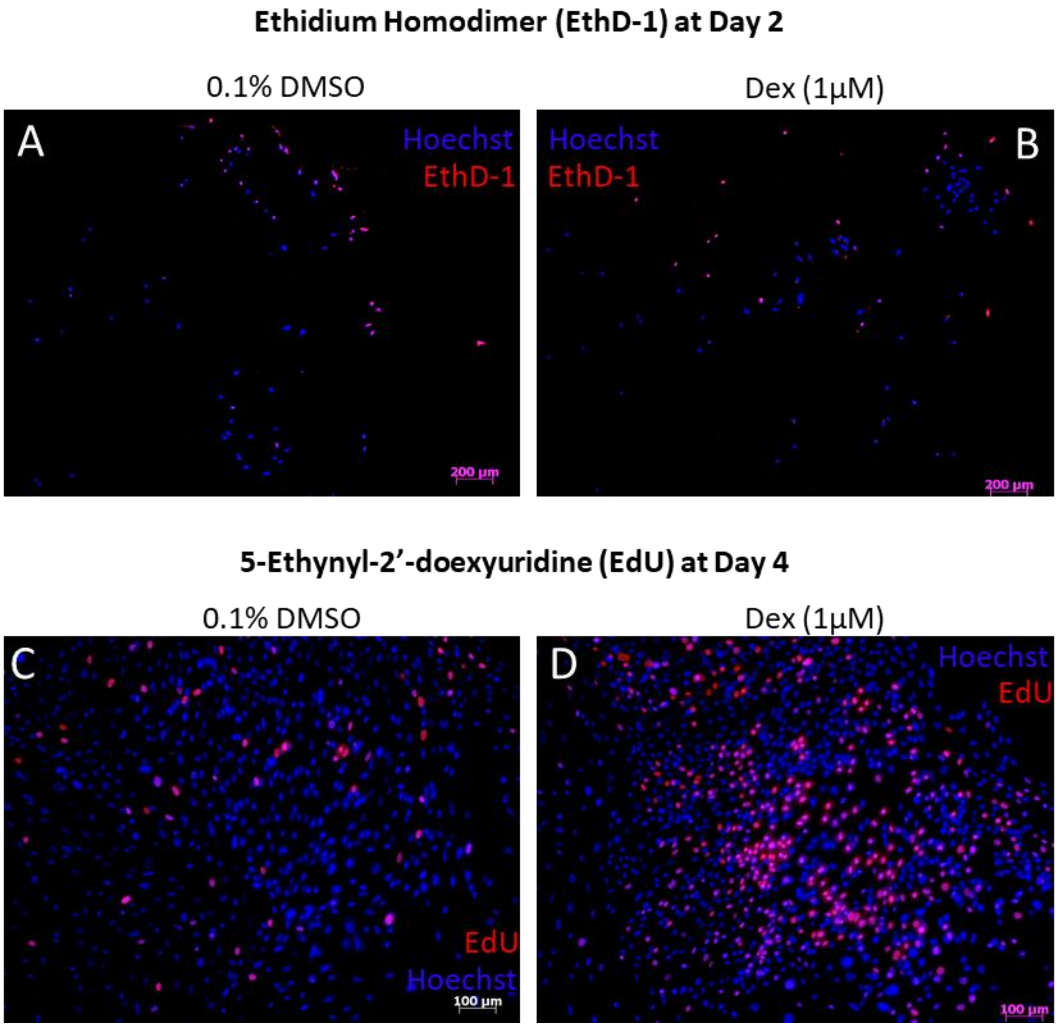

Figure S2. Representative staining of cell death marker ethidium homodimer (EthD-1) and thymidine analog EdU.

(A-B) EthD-1 labeling at Day 2 in cells treated with **(A)** 0.1% DMSO, or **(B)** 1µM Dexamethasone.

(C-D) EdU labeling at Day 4 in cells treated with **(A)** 0.1% DMSO, or **(B)** 1µM Dexamethasone.

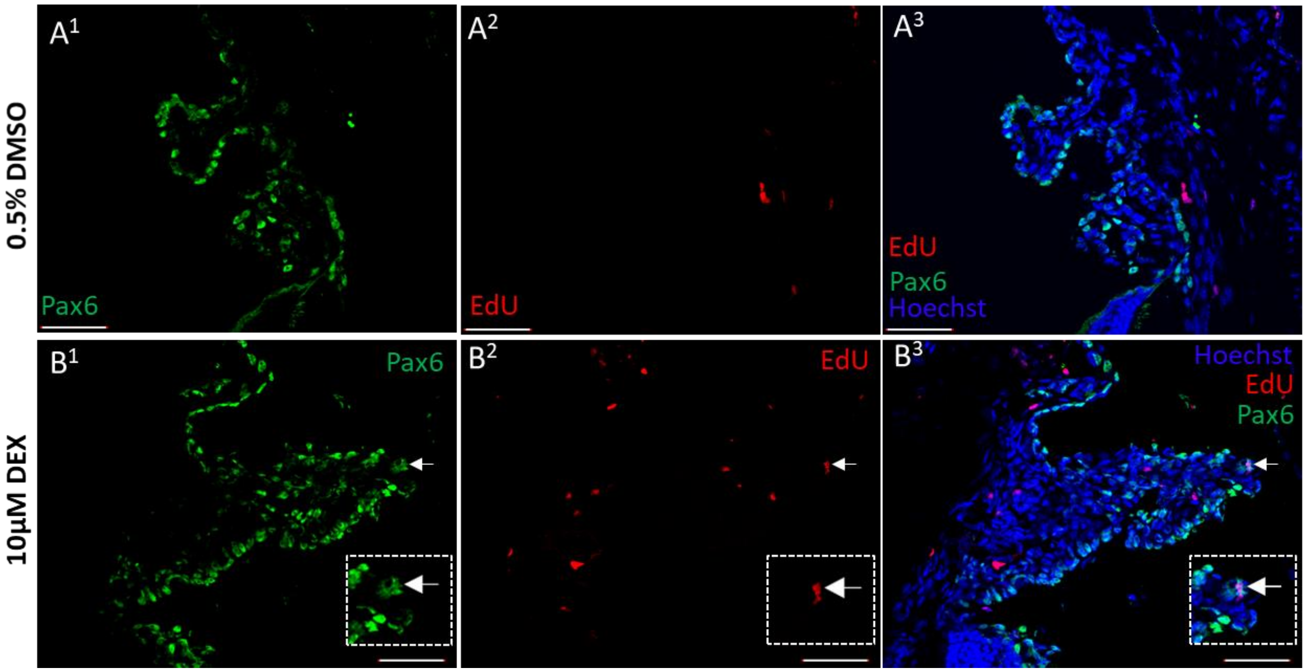

Figure S3. Intravitreal dexamethasone injection induces ciliary epithelium proliferation

(A-B) Representative images of Pax6 IHC and EdU labeling in the ciliary body of mouse eyes exposed to **(A)** 0.5% DMSO vehicle, or **(B)** 1µM Dexamethasone. Nuclei are labeled via Hoechst staining. White arrows indicate Pax6 + EdU co-labeled cells. Dashed line indicates inset. 10 µm-thick sections.

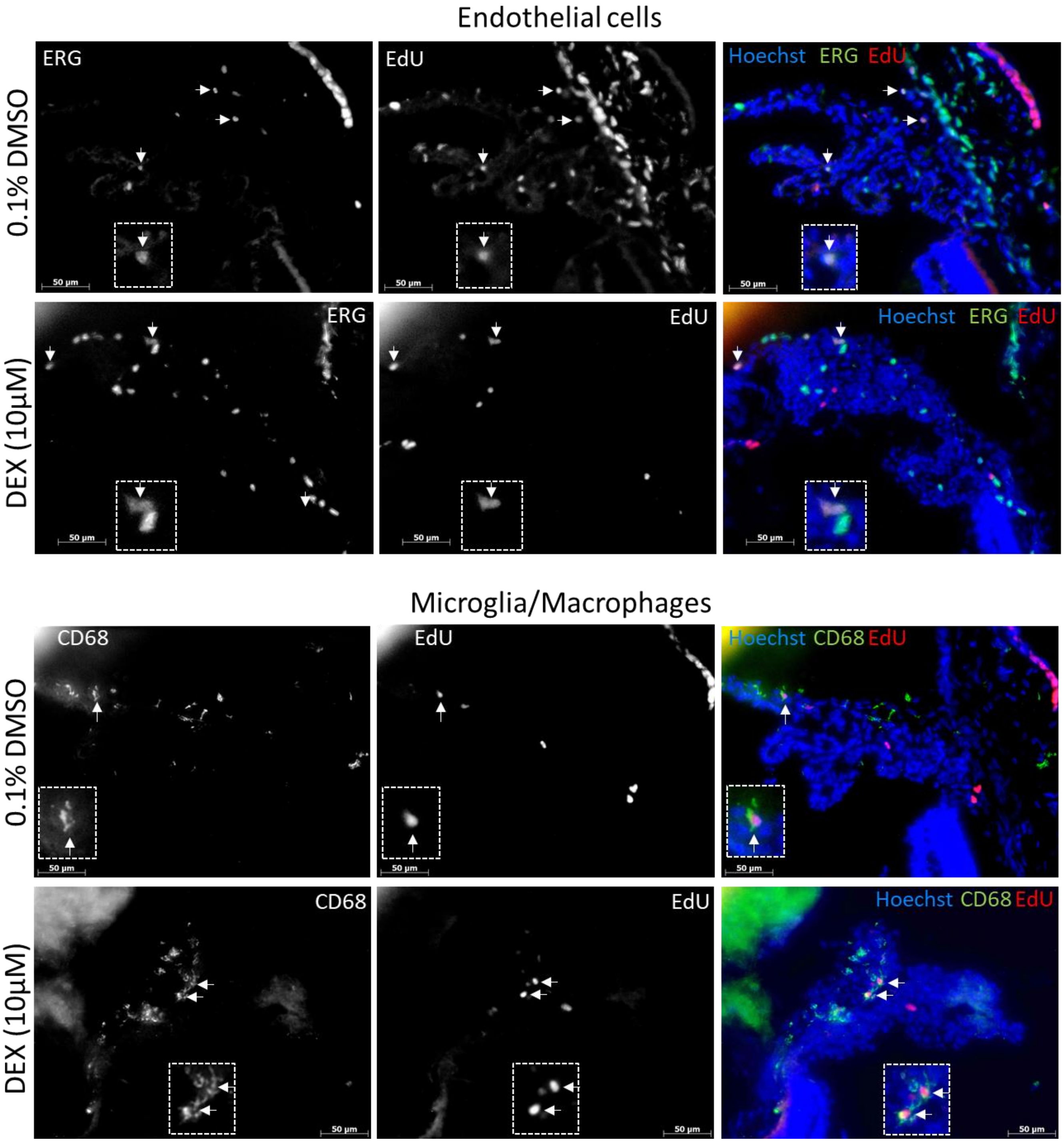

Figure S4. EdU-positive cells co-label with endothelial and microglia/macrophage markers

(A-B) Representative images of ERG IHC and EdU labeling in the ciliary body of mouse eyes exposed to **(A)** 0.5% DMSO vehicle, or **(B)** 1μM Dexamethasone.

(C-D) Representative images of CD68 IHC and EdU labeling in the ciliary body of mouse eyes exposed to **(C)** 0.5% DMSO vehicle, or **(D)** 1μM Dexamethasone. Nuclei are labeled via Hoechst staining. White arrows indicate co-labeled cells. 10 μm-thick sections.

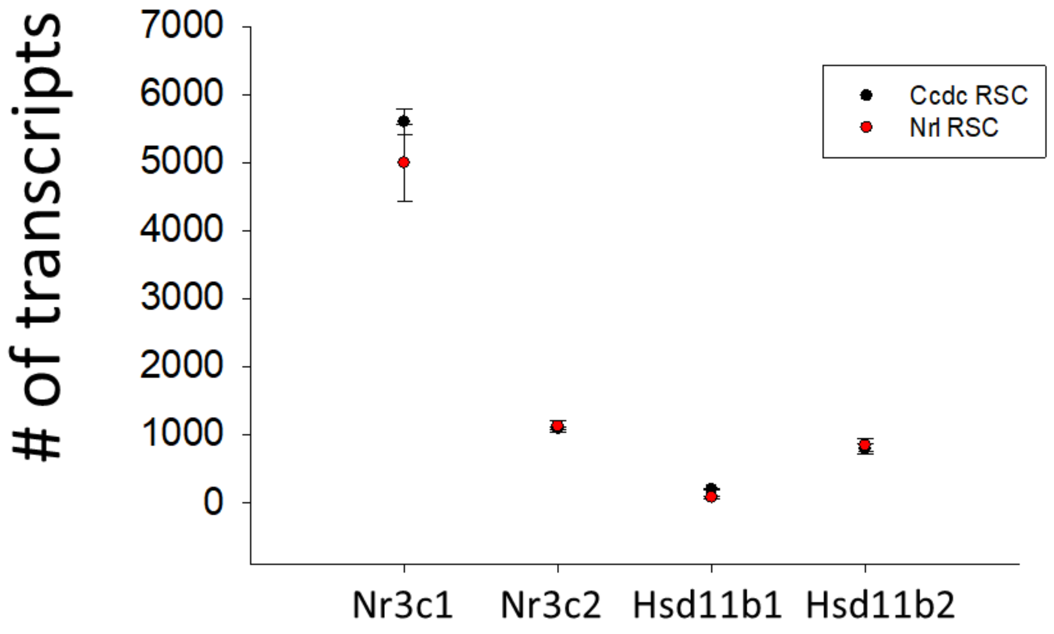

Figure S5. Glucocorticoid receptor, Mineralocorticoid receptor, and 11-β-HSD1 & 2 RNA expression in RSC spheres

Transcriptomic data showing the expression of the glucocorticoid receptor (Nr3c1), mineralocorticoid receptor (Nr3c2) and the two 11-β-HSD isozymes in RSC spheres, supporting the finding of retinal precursor sensitivity to GR agonists. This graph was created from RNAseq data collected in Khalili et al. 2018. Two different mouse strains were used to generate RSC spheres that were lysed and high-quality total RNA (RIN: 9–10) was subjected to directional RNA-sequencing library construction from three independent biological replicates per mouse strain. Sequencing was performed using GAIIx (Illumina, Inc., San Diego, CA; www.illumina. com).

SUPPLEMENTARY TABLES

Table S1. Compounds that met hit criteria in at least one of two screens

		Mechanism Hit Frequency		Screen 1 Hit			Screen 2 Hit		
**Compound**	**Mechanism**	**Freq.**	**P<0.05**	**Nuclei**	**GFP**	**Ratio**	**Nuclei**	**GFP**	**Ratio**
Prednisolone	Glucocorticoid receptors alpha and beta agonist	2/4	√	145%	160%	1.10	158%	162%	1.02
Dexamethasone	Glucocorticoid receptors alpha and beta agonist	2/4	√	142%	150%	1.05			
MK-0431	Dipeptidyl peptidase IV (CD26; DPP-IV) inhibitor	1/1	√	150%	137%	1.09			
MDV-3100	Androgen recepptor antagonist	1/5		139%	189%	1.36	*	*	
Abbvie Mcl-1 Cpd 30b	Mcl-1 inhibitor	1/5		140%	148%	1.06			
Sodium Butyrate	HDAC inhibitor	1/47		134%	208%	1.55	*	*	
Odanacatib	Cathepsin K inhibitor	1/1	√	136%	240%	1.76			
INCB024360	Indoleamine 2,3-dioxygenase inhibitor	1/1	√				143	139%	0.97
Thiazovivin	Rho/ROCK inhibitor	1/3					140%	132%	0.94
Lopinavir	HIV protease inhibitor	1/1	√				129%	254%	1.97
Guanabenz Acetate	A2-adrenergic receptor agonist	1/1	√				129%	142%	1.10
Irrestatin 9389	IRE1 inhibitor	1/3					137%	143%	1.04

* = seeding error: compound not in second screen

List of hit compounds and their known signaling pathways/mechanistic targets. Mechanism hit frequency indicates the number of hit compounds with the indicated mechanistic target relative to the total number of compounds with that target in the library. P-values are indicated for any target pathway with a significant enrichment within the library, as determined by the hypergeometric test. Percentages represent the number of nuclei or total actin-GFP area relative to 0.1% DMSO control (set as 100%). Percent of control is only indicated if a compound was found to be greater than three standard deviations above the control mean for both nuclei number and GFP area within a single screen. Ratio indicates the GFP area relative to the number of nuclei. A large GFP/Nuclei ratio indicates a disproportionate increase in cell area vs cell number and may signify compounds that caused cell hypertrophy.

Table S2. Screening quality metrics

	Screen 1		Screen 2	
	**Nuclei**	**GFP**	**Nuclei**	**GFP**
**SSMD**	-4.30 (Strong)	-2.83 (Moderate)	-3.75 (Strong)	-2.55 (Moderate)
**Coefficient of Variation**	13.92	11.51	7.97	10.21
**Signal:Noise**	8.54	10.9	18.08	4.16

Statistical evaluation of the variability and sensitivity of the MTS assay. Controls were determined to meet a threshold of statistical confidence to reliably determine hit compounds by assessing the variability within the 1x Control (coefficient of variation) and comparing the 1x Control with the pseudo-positive 2x Control (signal:noise ratio; strictly standardized mean difference (SSMD)). If SSMD is ≤ -2 and > -3 it is considered a moderate control. If SSMD is ≤ -3 and > -5 it is considered a strong control.
